# A Comparative Assessment of Nanotoxicity Induced by Metal (Silver, Nickel) and Metal Oxide (Cobalt, Chromium) Nanoparticles in *Labeo rohita*

**DOI:** 10.3390/nano9020309

**Published:** 2019-02-25

**Authors:** Zakia Kanwal, Muhammad Akram Raza, Farkhanda Manzoor, Saira Riaz, Ghazala Jabeen, Shafaq Fatima, Shahzad Naseem

**Affiliations:** 1 Department of Zoology, Faculty of Natural Science, Lahore College for Women University, Jail Road Lahore 54000, Pakistan; zakia.kanwal@lcwu.edu.pk (Z.K.); drfarkhanda786@gmail.com (F.M.); drghazala.jabeen@gmail.com (G.J.); shafaq.fatima@y7mail.com (S.F.); 2 Centre of Excellence in Solid State Physics, University of the Punjab, Quaid-e-Azam Campus, Lahore 54590, Pakistan; saira.cssp@pu.edu.pk (S.R.); shahzad.cssp@pu.edu.pk (S.N.)

**Keywords:** nanoparticles, nanotoxicity, *Labeo rohita*, sub-chronic exposure, biomolecules

## Abstract

In the present in vivo study, we provide a comparison of toxicological consequences induced by four different types of spherical nanoparticles (NPs)—silver nanoparticles (AgNPs, 40 ± 6 nm), nickel (NiNPs, 43 ± 6 nm), cobalt oxide (Co_3_O_4_NPs, 60 ± 6 nm), and chromium oxide (Cr_3_O_4_NPs, 50 ± 5 nm)—on freshwater fish *Labeo rohita*. Fish were exposed to NPs (25 mg/L) for 21 days. We observed a NPs type-dependent toxicity in fish. An altered behavior showing signs of stress and a substantial reduction in total leukocyte count was noticed in all NP-treated groups. A low total erythrocyte count in all NP-treated fish except for Co_3_O_4_NPs was discerned while a low survival rate in the case of Cr_3_O_4_NP-treated fish was observed. A significant decrease in growth and hemoglobin were noticed in NiNP- and Cr_3_O_4_NP-treated fish. A considerable total protein elevation was detected in NiNP-, Co_3_O_4_NP-, and Cr_3_O_4_NP-treated groups. An upgrading in albumin level was witnessed in Co_3_O_4_NP- and Cr_3_O_4_NP-treated groups while a high level of globulin was noted in NiNP- and Co_3_O_4_NP-exposed groups. In all NP-treated groups, a depleted activity of antioxidative enzymes and pathological lesions in liver and kidney were noticed.

## 1. Introduction

The exponentially growing demand of nanoparticles (NPs) in numerous applications is due to their incredible, exceptional, and astonishing properties that make them different to bulk materials [1]. The quantum confinement effects and large available active surface area of NPs are believed to be the key parameters that provide them with such outstanding physicochemical characteristics [2]. Nanotechnology has revolutionized the world by enhancing the efficiency and durability of nanostructure-based products. Presently, NPs are used widely in different industries, including agriculture, mechanical, food, energy, and electronics. Moreover, in modern chemistry and biomedical science, NPs are being successfully used in several applications [3].

Metallic NPs comprise a versatile class of materials including pure metal NPs—for example silver, gold, iron, cobalt, and nickel—and their compounds—such as oxides, hydroxides, sulfides, phosphates, fluorides, and chlorides [4]. The exciting surface plasmon properties, attractive physicochemical features, and distinct shape and size of metallic NPs make them potential candidates for memory storage devices, manufacturing of magnetic ferrofluids, photography, catalysis, photonics, and optoelectronics applications [5]. They are also found to be noteworthy in various medical diagnostic and therapeutic applications, e.g., biomagnetic separation and detection systems, magnetic gene transfection, stem cell engineering, immunoassay, and for immuno/aptasensors as platforms to immobilize biocompounds [6,7,8]. Moreover, antibacterial properties and the ability to be conjugated with different drugs, ligands, and antibodies proved metallic NPs to be successful antimicrobial agents against multi-drug-resistant organisms and as efficient nanocontainers for precise drug and gene transport [9,10,11].

The availability of more than 600 NP-based consumer products in the market reflects a drastic increase in the production of daily-use items with omnipresence of NPs. These products can be divided into eight categories: 1) household appliance; 2) health and fitness goods, such as sporting stuffs, sunscreen, and makeups; 3) food and beverage; 4) home and garden stuff including construction materials, home furnishings, and antibacterial paints; 5) automotive parts such as carbon nanotube spiked tires; 6) electronics and computers; 7) cross-cutting coatings; and 8) goods for children, such as silver NP-coated children’s toys etc. [12,13].

The growing and widespread applications of NPs have also led to concerns regarding their negative impact on human as well as environment health. Thus, nanotechnology can be considered to be a double-edged sword [14]. The released NPs from NP-based items will end up in the atmosphere, water, or soil, when disposed by any means. The exposure of released NPs to biological organisms can be by direct and indirect means. The possible indirect ways include inhalation or digestion for land-dwelling organisms, and through plants. The direct passage for aquatic organisms is likely through external surface epithelia or gills. When NPs are exposed to the environment, interaction of NPs with the surrounding materials and complex organic fluids could result the creation of corona. The produced corona can affect different functions of biological systems [15,16,17]. Furthermore, the reactivity, mobility, and biological fate of NPs are found to be size-, shape-, and charge-dependent.

For the formation of NPs of different size, shape, and composition, various chemical, physical, and biological approaches can be used, such as microemulsion, electrochemical synthetic approach, microwave-assisted synthesis photoinduced reduction, wet-chemical reduction, laser ablation, physical vapor condensation, and green synthesis-based approaches [2,18]. However, wet-chemical reduction method, owing to its easy handling, thermal stability, cost-effectiveness, high purity factor, and a variety of easily available chemicals, can be considered to be one of the best approaches. Different factors, including solvent, precursor, stabilizer, reducing agent, pH adjuster, and temperature, strongly influence the nucleation and growth processes occurring during reduction method. The appropriate use of these factors allows the synthesis of a wide range of NPs with controlled size, shape, and composition [19].

Nanotoxicity is the most prominent adverse effect caused by NPs when they interact with the environment and living systems. The exposure of NPs to living organisms induces various types of toxic attributes such as cytotoxicity (causing apoptosis, autophagy, and mitoptosis), genotoxicity (resulting mutagenicity, clastogenicity, and aneugenicity), and epigeneticity [20,21].

Changes in hematological, biochemical, oxidative enzyme, and histopathological parameters are considered to be the potential biomarkers for evaluating the toxicological impacts of NPs in living organisms. Moreover, owing to the evolutionary resemblances in the immune system of fish and humans, the fish model can mimic the biological responses happening in human well. Previous literature has also reported fish as an appropriate biological model to examine the toxicity caused by NPs. Verma et al. [22] used zebrafish (*Danio rerio*) to investigate toxic influences of industrially produced TiO_2_ NPs. They found that TiO_2_ NPs triggered cytotoxicity and caused alterations in ROS and neutral lipids. Griffitt et al. [23] noticed lethality and gill damage in zebrafish caused by copper NP exposure. Similarly, zebrafish was also used to analyze the damaging effects of SiO_2_ NPs on DNA strand break and antioxidant enzymes [24]. Magnetic iron oxide (Fe_2_O_3_) NPs caused chronic toxicity in *Labeo rohita* (*L. rohita*) by disturbing gill activity, hematology, and ion regulation [1]. Likewise, *L. rohita* was also employed as the fish model to evaluate antioxidative responses in gill, liver, and muscle induced by acute toxicity of AgNPs [3].

Using fish as a model organism also enhances its importance, because the marine atmosphere is specifically in danger due to NP exposure because water bodies serve as the final destination for the majority of environmental contaminants. It is, therefore, vital to examine biological responses of aquatic organisms caused by NPs. Although various studies on toxicity of nanomaterials have been carried out, in most of the investigations only one or two types of NP were used. So, it is indispensable to assess the nanotoxicity of the various magnetic and non-magnetic NPs comprehensively and comparatively to frame the safety measurements. Here we used *L. rohita* to ascertain a comparative analysis of the toxicological effects of silver, nickel, cobalt oxide, and chromium oxide NPs on hematology, biochemical, antoxidant defense system, and histopathological parameters. Our findings will provide an insight into potential toxicities of these NPs and health conditions of the treated fish.

## 2. Materials and Methods

### 2.1. Synthesis of Different Types of Metallic NPs

All chemicals used—nickel chloride hexahydrate (NiCl_2_·6H_2_O, 98% pure), tri-sodium citrate (Na_3_C_6_H_5_O_7_), cobalt chloride hexahydrate (CoCl_2_·6H_2_O), silver nitrate (AgNO_3_), sodium hydroxide (NaOH),ethylene glycol (C_2_H_6_O_2_, 99% PS), sodium borohydride (NaBH_4_), chromium chloride hexahydrate (CrCl_3_·6H_2_O), ethylenediaminetetra acidic acid (ETDA), and hydrazine hydrate (H_6_N_2_O)—were of analytical grade from Merck (Darmstadt, Germany). For solution purposes, highly purified deionized water was employed.

Four types of metallic NPs were synthesized by solution-based chemical reduction approaches using respective metallic precursors and appropriate reducing agents; brief preparation details of each type are described below.

Silver nanoparticles (AgNPs) were prepared following the same procedure as our previous study [2]. Briefly, to a boiling aqueous solution (50 mL) of AgNO_3_ (1mM), 5 mL aqueous solution of tri-sodium citrate (TSC, 1%) was introduced dropwise and allowed to complete under vigorous stirring. The solution color changed from transparent to yellow to greenish yellow. The value of pH was found to be 6.3.

**Figure 1:**
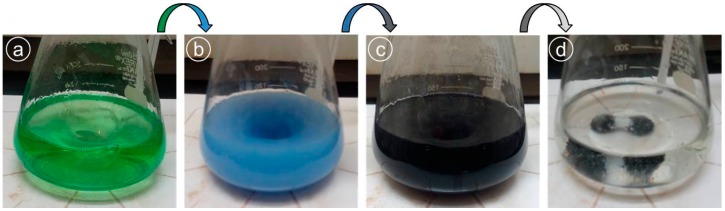
Different stages during the synthesis of NiNPs (**a**) color of nickel precursor solution, (**b**) hydrazine turned the color of solution to blue-violet, (**c**) solution color tuned to black afterwards, and (**d**) finally NiNPs recovered magnetically.

Nickel nanoparticles (NiNPs) were manufactured by a route described by Wu et al. [25]. Briefly, a precursor solution (pale green) was prepared at room temperature by mixing nickel chloride (1.0 g) into ethylene glycol (100 mL) under magnetic stirring (Figure 1a). The temperature was then raised to 60 °C and hydrazine (4.5 mL) was added, which turned the solution instantly to blue to blue-violet (Figure 1b) and after few seconds it turned into grey. The solution was kept for 2 min to homogenize itself and then NaOH (1M, 3 mL) was introduced to achieve the pH of 10 under continues stirring. The solution turned from grey to black (Figure 1c). After 60 min, heating and stirring was stopped to collect the prepared NPs magnetically.

Cobalt oxide nanoparticles (Co_3_O_4_NPs) were manufactured at room temperature by simple chemical reduction approach described elsewhere [26]. Firstly, an aqueous solution of TSC was prepared by dissolving 0.235 g of TSC into 10 mL deionized water under constant stirring (150 rpm). In the next step, 0.2 g of cobalt chloride and 0.1 g of NaBH_4_ were added simultaneously into the TSC solution at room temperature with continuing stirring (150 rpm). A huge amount of hydrogen was released and a boil in the solution was noticed. The stirring was turned off when no further hydrogen was releasing. The pH of the solution was found to be 8.0. Cobalt oxide NPs were collected by an external magnet in the form of greyish-black powder. The obtained powder was washed repeatedly with pure water and ethanol and allowed to dry for 24 h in air at room temperature.

Chromium oxide nanoparticles (Cr_3_O_4_NPs) were synthesized following Raza et al. [27] by solution-based chemical method using chromium chloride as a precursor and ethylene diamine tetra acidic acid (ETDA) as solvent. An appropriate amount of chromium chloride was dissolved into 0.2 M solution of ETDA at room temperature under constant stirring (200 rpm) for 30 min. Then, to adjust the solution pH to 9.0, sodium hydroxide was introduced to solution. The prepared NPs were obtained in the form of green precipitates after centrifuging at 4000 rpm for 60 sec. The centrifuged particles were dried at 400 °C for 60 min to obtain final product.

**Figure 2:**
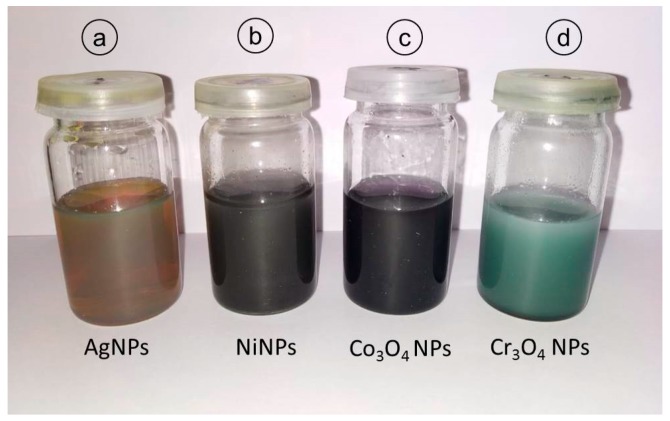
Pictures of synthesized colloidal samples showing the colors of final stage of solutions in each case. Different colors depict different composition of NPs.

The pictures of colloidal samples of all prepared NPs are presented in Figure 2 showing the final solution colors.

### 2.2. Characterization Techniques

To determine different characteristics of prepared NPs, various characterization techniques were employed. For optical absorption properties, a colloidal sample was characterized by UV-vis spectroscopy (Nicolet, Evolution 300, Thermo Electron Corporation, Waltham, MA, USA) in the 300–900 nm range of wavelength. Structural properties and crystallite size were measured by x-ray diffraction (XRD) techniques (D-maxIIA, Rigaku, Tokyo, Japan). For XRD measurement, a thick film of NPs was obtained by drying a few drops of colloidal sample on the clean glass substrate. The morphological analysis of the samples was conducted by scanning electron microscopy (SEM) (FEI Nova NanoSEM 450, Hillsboro, OR, USA). For the magnetic analysis of particles, a vibrating sample magnetometer (7407, Lakeshore, Westerville, OH, USA) was used.

### 2.3. Fish and Fish Care

*L. rohita* juveniles of average length 10.5 ± 1.3 cm and weight 21 ± 2.5 g were transported from Himalya fish hatchery, Murid Key, Lahore, Pakistan to the lab. Fish were kept in glass aquaria (100 L capacity) and were acclimatized in the lab for two weeks. Fish were handled in accordance to the local animal welfare regulations. During this period, each fish was fed 3% of its body weight daily with a basal diet comprising fish meal (28%); soybean meal (35%); maize (15%); wheat bran (10%); wheat (7%); vegetable oil (4%); vitamin premix (0.9%); and mineral premix (0.1%). To ensure optimal dissolved oxygen concentration, the water of the fish aquarium was exchanged daily. Aerators were kept fixed with the tanks to facilitate continuous oxygen supply. During treatment period, the measured physicochemical properties of the aquarium water were as below: pH 6.9–7.8; ammonia 0.1–0.28 mg/L; dissolved oxygen range 5.7–7.5 mg/L; chloride 10.2 mg/L; temperature variation 26.5–28 °C; and nitrate 0.02–0.09 mg/L.

### 2.4. NPs Exposure to Fish

Five groups were prepared by a random distribution of fish: control group (untreated), AgNP-exposed group, NiNP-exposed group, Co_3_O_4_NP-exposed group and Cr_3_O_4_NP-exposed group. All NP exposure experiments were performed in triplicate. Freshly prepared sonicated NPs were administered daily to the fish by mixing in tank water. A similar concentration of 25 mg/L of each type of NP was used for a 21-day exposure period. Survival and behavior of fish was monitored regularly during the experimental period.

### 2.5. Hematological Analysis

For hematological studies, clove oil (100 µg/L) was used to anesthetize the fish to collect blood from the caudal vein. Ethylenediaminetetra acetic acid (EDTA) was used as anticoagulant. Diluting medium (Turk’s solution and Toisson’s solution) was used for total leukocyte count (TLC) and total erythrocyte count (TEC) respectively. A hemocytometer was used for cell counting. Cyanmethemoglobin method using Drabkin’s fluid was followed for hemoglobin (Hb) detection, and spectrophotometer was used for absorbance (at 540 nm) measurements. Hematocrit (Hct %) measurements were conducted following the method defined by Dacie and Lewis [28] by using microhematocrit tubes.

### 2.6. Biochemical Analysis

After keeping the collected blood at room temperature for two hours, blood samples were centrifuged at 3000 rpm for 10 min. The obtained serum was used for further biochemical tests. Total protein was calculated by using total protein kit (Crescent Diagnostics, Jeddah, Saudi Arabia) which employs Photometric Colorimetric-Biuret method. Albumin was calculated by using albumin kit (Crescent Diagnostics, Jeddah, Saudi Arabia). For globulin calculation, the amount of Albumin was subtracted from Total protein. Enzymatic-colorimetric method was used for cholesterol measurements. Kinetic enzyme assays were used for alanine aminotransferase (ALT) and aspartate aminotransferase (AST) measurements.

### 2.7. Oxidative Stress Analysis

Oxidative stress analysis was performed on kidney and liver tissues. Liquid nitrogen was used for a snap-freezing process of the kidney and liver tissues, and subsequently samples were kept at –40 °C for further use. The frozen tissue samples were rinsed many times with chilled phosphate buffer solution (PBS). A Tenbroek glass homogenizer was used to homogenize the samples. Afterwards, tissue samples were centrifuged at a speed of 10,000 rpm for a duration of 15 min at a temperature of 4 °C. The level of malondialdehyde (MDA) and antioxidant enzymes—including superoxide dismutase (SOD), catalase (CAT), and glutathione peroxidase (GPx) activities—were measured in the supernatant by spectrometry [29]. Values of MDA, SOD, CAT, and GPx are measured as the amount of the molecules per milligram or gram of protein, and international units per milligram or gram of protein, respectively.

### 2.8. Histological Analysis

For histological analysis, tissues (kidney and liver) were fixed for 24 h in 10% buffered formalin to prevent autolysis of cellular morphology. Afterwards, fixation tissues were washed twice in phosphate buffered saline, dehydrated through a series of various concentrations of alcohol in ascending order (10–15 min each), and finally kept in 75% ethanol until processed further. Tissues were then embedded in paraffin wax. By using rotatory microtome (ERM-2301), sections of 7–8 µm were cut. The hematoxylin and eosin were used to stain the sliced sections. The stained sections were put on the glass slides and were examined with an optical microscope (Eclipse E-200 Microscope, Nikon, Tokyo, Japan). Photographs of each slide were taken at 10× and 40× magnifications.

### 2.9. Statistical Analysis

The collected data was analyzed statistically to determine significant differences between control and NP-treated groups. The average of replicates is taken as mean, and the error bars indicate the standard error to the mean. A one-way analysis of variance (ANOVA) followed by a Tukey’s multiple comparisons test was adopted to calculate the statistical significance in each case. GraphPad Prism (ver.7.03, San Diego, California, CA, USA) was used for all statistical tests.

## 3. Results

### 3.1. Characterization of Prepared NPs

The changes in the solution color during the synthesis of NPs by chemical reduction method indicate the occurring of different stages in the reaction. For example, in the case of NiNP preparation (Figure 1), the appearance of pale green color was due to the creation of nickel ions (Ni^+^) in the solution and alteration of pale green to blue-violet to grey and finally black was the indication of nickel ion (Ni^+^) reduction into free nickel atoms (Ni^0^) by gaining electrons. The free atoms gathered under the action of Brownian motion and van der Waals interactions to form Ni-nuclei (nucleation process) and their growth resulted in the NiNPs formation [19,25].

**Figure 3:**
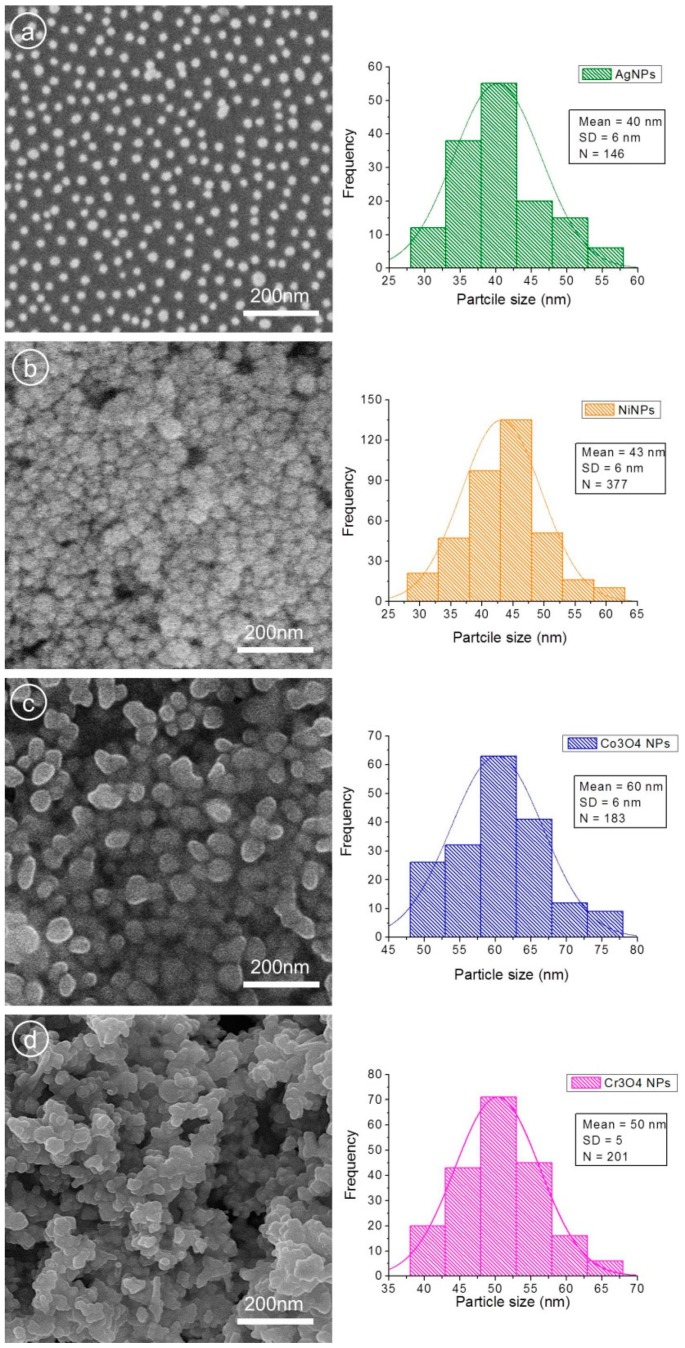
Right panel represents the SEM micrographs revealing the surface morphology, while left panel displays histograms showing the particle size distribution of prepared particles (**a**) AgNPs, spherical, with average particle size of 40 ± 6 nm, (**b**) NiNPs, spherical, with average diameter 43 ± 6 nm, (**c**) Co_3_O_4_NPs, spherical, with 60 ± 6 nm average dimeter, (**d**) Co_3_O_4_NPs, spherical and near spherical, with 50 ± 5 nm average size.

The structural, optical, magnetic, and morphological analyses of synthesized NPs were carried out by aforementioned characterization techniques. In Figure 3, SEM images (left panel) and size distribution histograms (right panel) with average particle size of all four NPs samples are presented. In the case of AgNPs (Figure 3a), nicely scattered spherical particles can be observed. Agglomeration of smaller particles in a few places can also be noticed. The average particle size of AgNPs was found to be 40 ± 6 nm as indicated in the histogram image (Figure 3a, left). The morphology of NiNPs is displayed in Figure 3b, where high density of particles with relatively spherical shapes with average diameters of 43 ± 6 nm can be observed. A high degree of aggregation of the individual NPs depicts the magnetic nature of NiNPs. SEM micrographs of cobalt oxide NPs (Figure 3c) show agglomerated and larger spherical particles with average size of 60 ± 6 nm, as displayed in the Co_3_O_4_NP histogram (Figure 3c, right). Chromium oxide NPs (Figure 3d) appeared spherical and nearly spherical in shape, and cluster formations can also be seen. The histogram of Cr_3_O_4_NPs (Figure 3d, right) displays an average particle size of 50 ± 5 nm.

**Figure 4:**
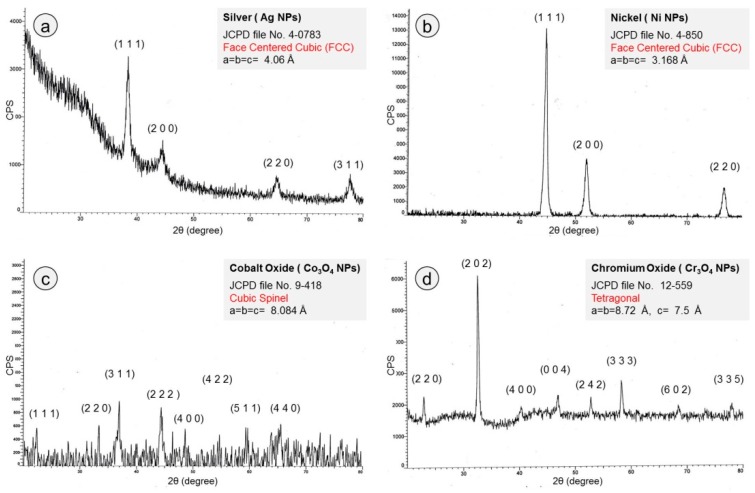
X-ray diffraction (XRD) spectra; (**a**) AgNPs, (**b**) NiNPs, (**c**) cobalt oxide NPs, and (**d**) chromium oxide NPs, indicating FCC crystalline metallic nature of AgNPs and NiNPs, cubic spinel crystalline structure of cobalt oxide NPs and tetragonal crystalline behavior of chromium oxide NPs.

The crystalline nature and phase purity of all prepared NPs were determined by using the XRD approach at room temperature and the obtained patterns are presented in Figure 4a–d. The prominent sharp peaks in each spectrum indicate the crystalline nature of the obtained metallic NPs.

In the case of AgNPs (Figure 3a), four reflection planes can be identified by four major characteristic peaks, such as (111) at 2*θ* = 38.5°, (200) at 2*θ* = 44.7°, (220) at 2*θ* = 64.7°, and (311) at 2*θ* = 77.6°. This XRD spectrum indicates that prepared NPs were of pure metallic silver nature with polycrystalline structure of face-centered cubic (FCC) according to JCPDS file No. 4-0784 [30]. The XRD spectrum for NiNPs is presented in Figure 4b, where three sharp distinct diffraction peaks at 44.8°, 51.9°, and 78.4° values of 2*θ* can clearly be observed. These peaks correspond to the (111), (200), (222), and (220) planes of crystalline FCC nickel according to JCPDS file No. 4-850 [25,31]. The XRD pattern of cobalt oxide NPs (Figure 4c), exhibits reflections planes (111), (220), (311), (222), (400), (422), (511), and (440) at 2*θ* = 22.7°, 33.4°, 36.9°, 44.5°, 48.7°, 54.7°, 59.3°, and 65.7° respectively, confirming the cubic spinel structure of Co_3_O_4_NPs according to JCPDS File No. 9-418 [32,33]. Figure 4d depicts the indexed XRD spectrum of synthesized Cr_3_O_4_NPs. The well-defined diffraction peaks appearing at 2θ values of 23.0°, 32.7°, 41.3°, 46.9°, 53°, 58.2°, 68.5°, and 78.0° can be assigned to reflection planes (220), (202), (400), (004), (242), (333), (602), and (335) respectively according to the JCPDS File No. 12-559 [34]. The obtained XRD results suggest that prepared particles were Cr_3_O_4_NPs with tetragonal crystalline phase.

Using the broadening of the most predominant orientation as (111) for silver and nickel NPs and (311) and (202) for cobalt oxide and chromium oxide NPs, the crystallite size (D) was estimated by following Scherer formula [35]:

(1) $$D=\delta \lambda /\beta_{hkl}cos\theta$$

where *δ*, *λ*, *β_hkl_*, and *θ* are shape factor (for spherical shapes = 0.9), x-ray wavelength (1.540 Å in this case), width of diffraction peak (full width at half maximum, FWHM), and diffraction angle, respectively. All measured values of different structural features are registered in Table 1.

**Figure 5:**
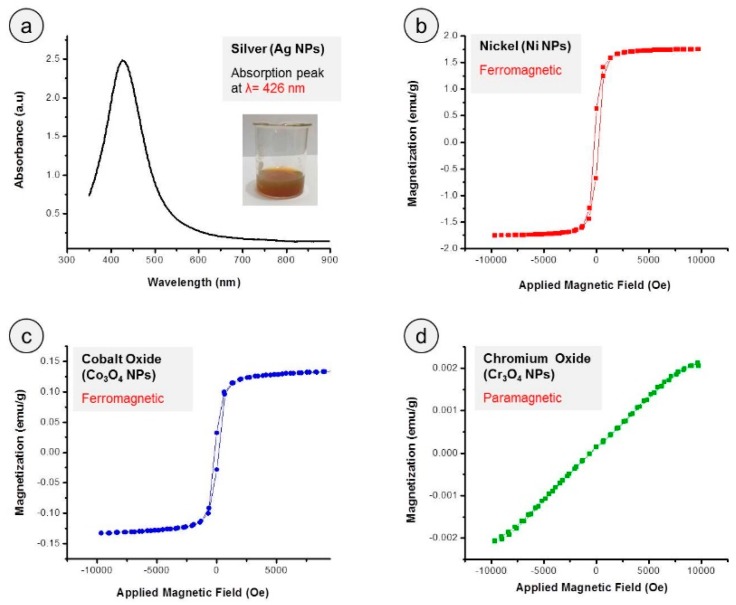
(**a**) UV-visible absorption spectra of AgNPs showing a single characteristic peak. The inset displays the color of colloidal AgNPs sample in a beaker. (**b**–**d**) Magnetic hysteresis loops of NiNPs, cobalt oxide NPs, and chromium oxide NPs demonstrating the ferromagnetic behavior of NiNPs and cobalt oxide NPs while paramagnetic nature of chromium oxide NPs.

The optical absorption spectrum of colloidal AgNPs sample obtained by UV-Visible spectrophotometer is presented in Figure 5a. The single characteristic absorption peak occurring at 426 nm (wavelength, *λ*) with FWHM value of about 107 nm confirmed the presence of spherical silver NPs with a wide range of size distribution. The vibrating sample magnetometer (VSM) measurements were conducted to determine magnetic properties of prepared nickel, cobalt oxide and chromium oxide NPs and obtained magnetic hysteresis loops are shown in Figure 5b–c. The VSM plots revealed the ferromagnetic behavior of nickel and cobalt oxide NPs, while chromium oxide NPs were found to be paramagnetic in nature. The VSM-measured magnetic results are recorded in Table 2.

### 3.2. Survival and Growth Studies

The survival of fish was noted in each group after NPs exposure. No fish mortality was observed in AgNP-, NiNP-, and Co_3_O_4_NP-exposed groups, and survival rate was 100% during the test period. However, in the case of Cr_3_O_4_NP-exposed fish group, a low percentage survival (86%) was noticed, which confirmed an acute lethal toxicity of Cr_3_O_4_NPs to fish. The obtained survival results are graphically presented in Figure 5a.

**Figure 6:**
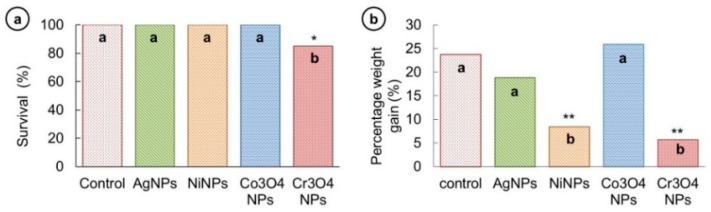
(**a**) Survival rate of fish in control, AgNP-, NiNP-, and Co_3_O_4_NP-exposed groups was 100%. The fish treated with Cr_3_O_4_NPs had a significantly low survival rate (86%). (**b**) Percentage weight gain was not significantly different among control, AgNP- and Co_3_O_4_NP-exposed fish while it was significantly lower in NiNP- and Cr_3_O_4_NP-exposed fish. Columns showing different letters (**a**,**b**) are significantly different, (* *p* < 0.05) (** *p* < 0.01).

The growth analysis of fish in all five groups was performed by measuring the percentage weight gain ((difference between final and initial weight/initial weight) × 100) and displayed in Figure 6b. Percentage weight gain was found not to be significantly different among control (23.7%), AgNP- (18.8%) and Co_3_O_4_NP-treated (25.9%) fish groups. A significant reduction in weight of fish treated with NiNPs (8.4%) and Cr_3_O_4_NPs (5.6%) indicates that these two types of NP have severely affected the growth of the treated fish.

### 3.3. Behavioral Changes

The behavior of fish was observed twice a day during the experimental period. Significant behavioral alterations were recorded in NP-treated fish groups. Four behavioral categories—swimming, interaction, fin movements, and feed intake—were documented and listed in Table 3. Fish in the Control group showed all normal activities. Fish treated with AgNPs did not show any swimming problems and had normal interaction with other fish. Fin movements and feed intake was also normal in this group. The NiNP-treated group showed disturbed swimming, irregular interactions, slow fin movements, and very low feed intake. Addition of Co_3_O_4_NPs into water caused a fast color change of the water from transparent to dark brown. Fish in the Co_3_O_4_NP-treated group showed random movements, avoiding behavior, lethargic fin movement, and low feed intake. A color change of the water from transparent to cloudy grey was observed when colloidal solution of Cr_3_O_4_NPs was introduced into the aquarium water. Fish treated with Cr_3_O_4_NPs showed restlessness, random swimming, avoiding and aggressive behavior, slow fin movements, and very little feed intake (Table 3).

### 3.4. Hematological Responses

**Figure 7:**
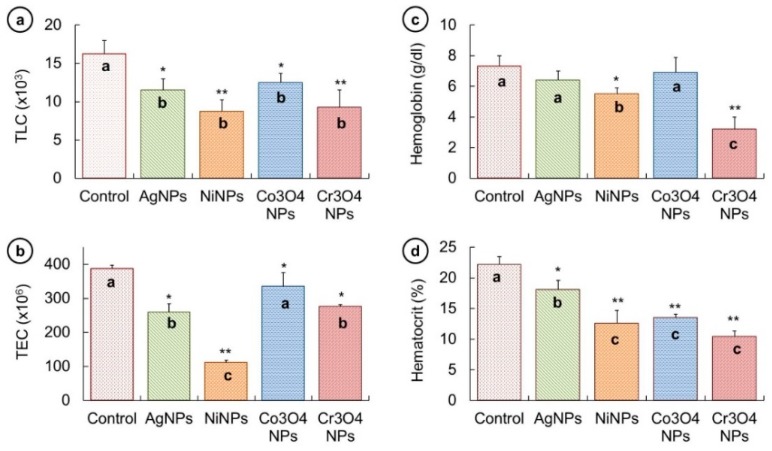
(**a**) Total number of leukocytes (TLC) was significantly lower in all fish groups treated with NPs than the control group. (**b**) The total number of erythrocytes (TEC) was not significantly different in control and Co_3_O_4_NP-treated groups but was significantly decreased in other groups. (**c**) The hemoglobin content was significantly reduced in NiNPs and Cr_3_O_4_NP-treated groups. (**d**) The hematocrit % was significantly dropped in all NP-treated groups. Columns showing different letters (a, b, and c) are significantly different, (* *p* < 0.05) (** *p* < 0.01).

The variation in different hematological parameters such as TLC, TEC, Hb, and Hct % were measured and a comparison among all groups was made. All the obtained values are demonstrated in graphical form in Figure 7.

It can be observed from Figure 7a that TLC was reduced in all fish groups treated with different NPs in comparison with the untreated fish. This reduction can be listed as control (16.2 ± 1.7 × 10^3^) > Co_3_O_4_NPs (12.5 ± 1.2 × 10^3^) > AgNPs (11.5 ± 1.5 × 10^3^) > Cr_3_O_4_NPs (9.2 ± 2.2 × 10^3^) > NiNPs (8.7 ± 1.5 × 10^3^). This sequence indicates that NiNPs proved more toxic in affecting the TLC. The analysis of TEC values shows no significant change in untreated and Co_3_O_4_NP-treated fish; however, fish treated with AgNPs, NiNPs, and Cr_3_O_4_NPs showed significantly lower TEC than that of the control fish (Figure 7b). The change in TEC can be ordered as control group (387 ± 10.6 × 10^6^) > Co_3_O_4_NPs (335 ± 40 × 10^6^) > Cr_3_O_4_NPs (276 ± 4.7 × 10^6^) > AgNPs (259.1 ± 24 × 10^6^) > NiNPs (111.05 ± 7.7 × 10^6^). Again, the NiNPs were observed more noxious to disturb the TEC.

The hemoglobin content was also found to be reduced in fish treated with metallic NPs, as depicted in Figure 7c. It was noticed that the decline in Hb was not significant in fish exposed to AgNPs and Co_3_O_4_NPs, but it was significantly reduced in fish groups treated with NiNPs and Cr_3_O_4_NPs than that of the untreated fish. The reduction in Hb in NP-treated fish groups can be put in a sequence as control (7.3 ± 0.7 g/dL) > Co_3_O_4_NPs (6.9 ± 1 g/dL) > AgNPs (6.4 ± 0.6 g/dL) > NiNPs (5.5 ± 0.4 g/dL) > Cr_3_O_4_NPs (3.2 ± 0.8 g/dL). A similar trend in Hct % response was witnessed and displayed in Figure 7d; it can be observed that significantly lower Hct % values were recorded in NP-treated groups. The reduction in Hct % was found in the following order: control (22.2 ± 1.3%) > AgNPs (18.1 ± 1.5%) > Co_3_O_4_NPs (13.5 ± 0.6%) > NiNPs (12.6 ± 2.1%) > Cr_3_O_4_NPs (10.4 ± 0.9%).

### 3.5. Biochemical Evaluation

**Figure 8:**
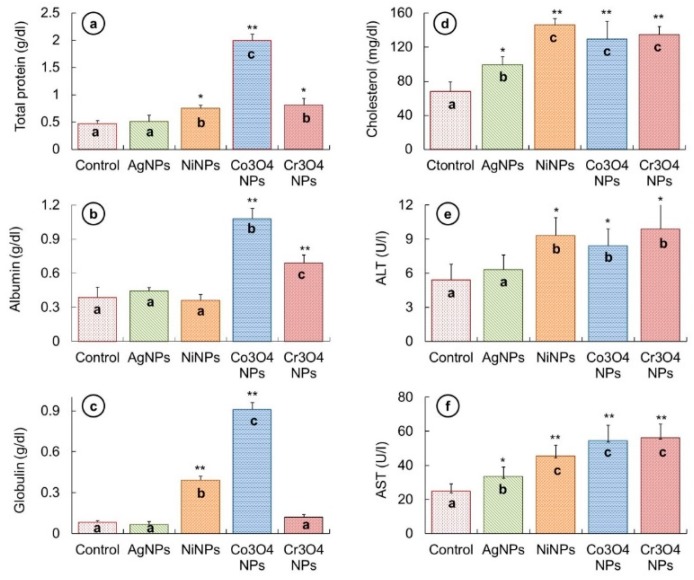
(**a**) Total protein was found to be significantly high in NiNP-, Co_3_O_4_NP-, and Cr_3_O_4_NP-treated fish. (**b**) Albumin was significantly high in Co_3_O_4_NP- and Cr_3_O_4_NP-treated fish groups in comparison to the control. (**c**) The globulin value was significantly high in NiNP- and Co_3_O_4_NP-treated fish than control. (**d**) Cholesterol level was significantly high in all treated groups. (**e**) ALT concentration was significantly high in NiNP-, Co_3_O_4_NP-, and Cr_3_O_4_NP-treated groups in comparison to the control. (**f**) Concentration of AST was significantly high in all treated groups than control. Columns showing different letters (a, b, and c) are significantly different, (* *p* < 0.05) (** *p* < 0.01).

To investigate the biochemical effects caused by NP treatment in fish, variations occurred in total protein, albumin, globulin, cholesterol, ALT, and AST activities were measured from the serum and compared with that of the control fish. The measured values are shown in Figure 8.

An increase in the amount of total protein was noticed in all fish groups treated with NPs, as demonstrated in Figure 8a. The increase in AgNP-treated fish (0.51 ± 0.12 g/dL) was not significantly different to that of untreated fish (0.46 ± 0.06 g/dL). However, the elevation in total protein levels was significantly high in the other three NP-treated fish groups in the following order Co_3_O_4_NPs (1.99 ± 0.12 g/dL) > Cr_3_O_4_NPs (0.81 ± 0.13 g/dL) > NiNPs (0.75 ± 0.06 g/dL).

In the case of albumin level variations (Figure 7b), the difference in the albumin values of AgNP- (0.44 ± 0.03 g/dL) and NiNP-treated (0.36 ± 0.05 g/dL) fish groups was not significant as compared to untreated fish group (0.38 ± 0.09 g/dL). Nevertheless, the value of albumin was significantly high in Co_3_O_4_NPs (1.08 ± 0.09 g/dL) and Cr_3_O_4_NPs (0.69 ± 0.07 g/dL) exposed fish.

The alterations in the globulin values (Figure 8c) revealed a significantly increased level of globulin in fish exposed to NiNPs (0.39 ± 0.03 g/dL) and Co_3_O_4_NPs (0.91 ± 0.05 g/dL), while no significant change was noticed in AgNP- (0.06 ± 0.02 g/dL) and Cr_3_O_4_NP-treated (0.12 ± 0.02 g/dL) fish samples as compared to the control fish samples (0.08 ± 0.01 g/dL). The concentration analysis of serum cholesterol indicated a significant increase in cholesterol level in all samples of fish exposed to NP in comparison with unexposed fish (Figure 8d) in the following order; NiNPs (145.9 ± 8 mg/dL) > Cr_3_O_4_NPs (134.4 ± 10 mg/dL) > Co_3_O_4_NPs (129.1 ± 21 mg/dL) > AgNPs (99.2 ± 10 mg/dL) > control (67.6 ± 12 mg/dL).

An increasing trend was recorded in both ALT and AST levels in all fish treated with NPs than that of untreated fish (Figure 8e,f). ALT activity was observed to be significantly high in fish exposed to NiNPs (9.3 ± 1.6 U/L), Co_3_O_4_NPs (8.4 ± 1.5 U/L), and Cr_3_O_4_NPs (9.9 ± 2.3 U/L); however, the increase in the case of AgNPs (6.3 ± 1.3 U/L) was not significant as compared to the untreated fish (5.4 ± 1.4 U/L). AST activity was noticed to be affected significantly in all NP-treated groups in the following sequence; Cr_3_O_4_NPs (56.3 ± 7.8 U/L) > Co_3_O_4_NPs (54.6 ± 9 U/L) > NiNPs (45.4 ± 6.3 U/L) > AgNPs (33.5 ± 5.5 U/L) > control (24.8 ± 4.4 U/L).

### 3.6. Oxidative Stress

**Figure 9:**
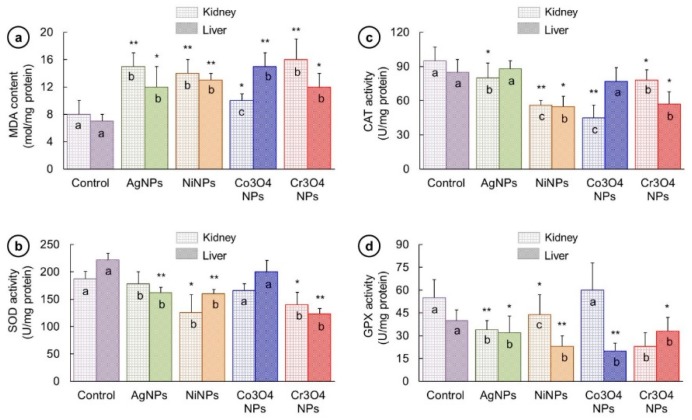
(**a**) Malondialdehyde (MDA) was observed to be significantly high in kidney and liver of all NP-treated groups (**b**) Superoxide dismutase (SOD) activity was significantly low in kidney of NiNP- and Cr_3_O_4_NP-treated groups and liver of AgNP-, NiNP-, and Cr_3_O_4_NP-treated groups. (**c**) Catalase (CAT) activity was significantly low in NiNP- and Cr_3_O_4_NP-treated kidney and NiNP- and Cr_3_O_4_NP-treated liver. (**d**) Glutathione peroxidase (GPx) activity was significantly low in AgNP-, NiNP-, and Cr_3_O_4_NP-treated kidney and the liver of all treated groups. Columns showing different letters (a, b, and c) are significantly different. (* *p* < 0.05) (** *p* < 0.01).

The level of oxidative stress marker (MDA) and antioxidant enzymes (SOD, CAT, GPx) were assessed in kidney and liver, and the obtained results are exhibited in Figure 9. The administration of NPs caused elevation in MDA content (Figure 9a). The values of MDA were found to be elevated both in kidney and liver of all NP-treated groups compared to that of control in the following sequence in kidney: Cr_3_O_4_NPs (16 ± 3 mol/mg) > AgNPs (15 ± 2 mol/mg) > NiNPs (14 ± 2 mol/mg) > Co_3_O_4_NPs (10 ± 1 mol/mg) and in liver this order was Co_3_O_4_NPs (15 ± 2 mol/mg) > NiNPs (13 ± 1 mol/mg) > Cr_3_O_4_NPs (12 ± 2 mol/mg) / AgNPs (12 ± 3 mol/mg). However, there occurred a decline in the activities of SOD, CAT, and GPx enzymes (Figure 9b–d). In case of SOD activity (Figure 9b) this decreasing severity was in the following order in kidney: AgNPs (178 ± 22 U/mg) > Co_3_O_4_NPs (166 ± 12 U/mg) > Cr_3_O_4_NPs (140 ± 23 U/mg) > NiNPs (126 ± 32 U/mg) and in liver: Co_3_O_4_NPs (200 ± 21 U/mg) > AgNPs (162 ± 10 U/mg) > NiNPs (160 ± 8 U/mg) > Cr_3_O_4_NPs (123 ± 10 U/mg). Down-regulation of CAT activity (Figure 9c) in kidney was observed in the following way: AgNPs (80 ± 13 U/mg) > Cr_3_O_4_NPs (78 ± 9 U/mg) > NiNPs (56 ± 4 U/mg) > Co_3_O_4_NPs (45 ± 11 U/mg) while in liver the order was: AgNPs (88 ± 7 U/mg) > Co_3_O_4_NPs (77 ± 12 U/mg) Cr_3_O_4_NPs (57 ± 11 U/mg) > NiNPs (55 ± 9 U/mg). A slight increase in CAT activity was recorded in AgNP-treated fish liver but this was not significant. The GPx activity (Figure 9d) in Co_3_O_4_NP-treated kidney was observed slightly higher (60 ± 18 U/mg) than that of unexposed fish kidney (55 ± 12 U/mg); nevertheless, in other three groups the activity was suppressed in the following sequence NiNPs (44 ± 13 U/mg) > AgNPs (34 ± 6 U/mg) > Cr_3_O_4_NPs (23 ± 9 U/mg). However, the GPx activity decreased in all NP-treated liver samples in the following order: Cr_3_O_4_NPs (33 ± 9 U/mg) > AgNPs (32 ± 11 U/mg) > NiNPs (23 ± 7 U/mg) > Co_3_O_4_NPs (20 ± 5 U/mg).

### 3.7. Histological Studies

**Figure 10:**
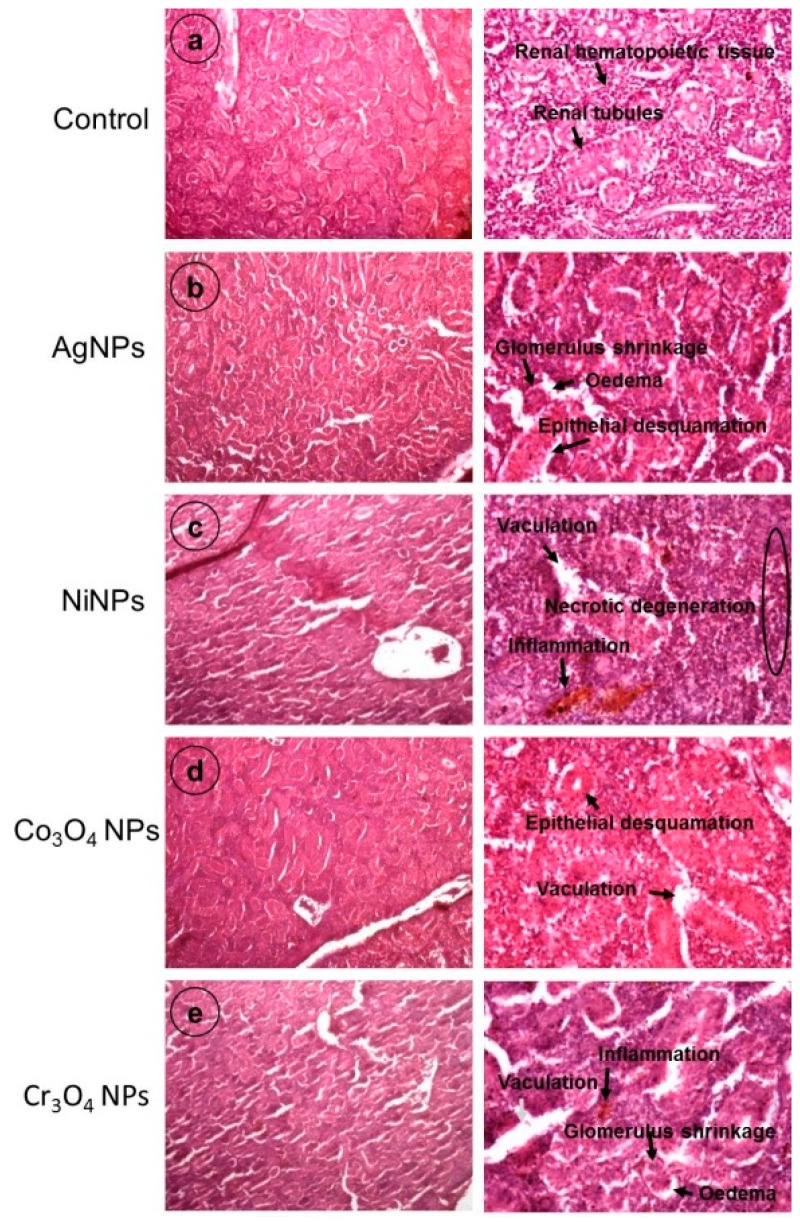
Histological evaluation of kidney of control and NP-treated fish groups demonstrating the alterations appeared in different parts caused by NPs exposure. Images are taken at two different magnifications: 10× (left panel); 40× (right panel).

**Figure 11:**
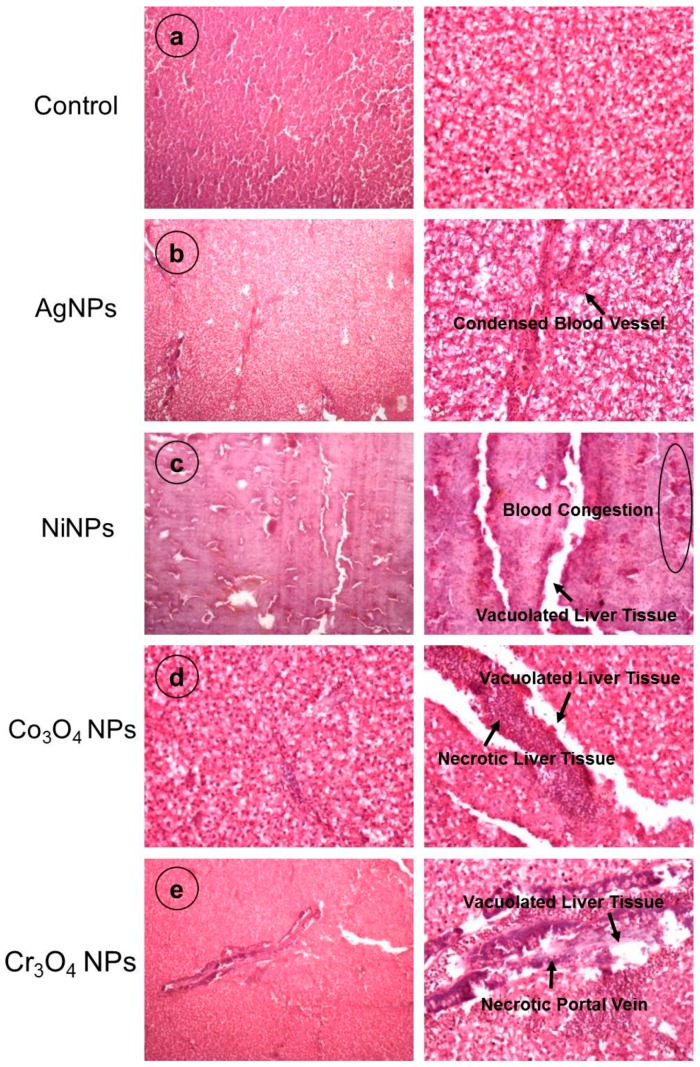
Histological examination of liver of fish exposed to NPs in comparison with control fish showing different types of damage caused by NPs treatment. Magnifications: 10× (left side), 40× (right side).

The histopathological observations were made for kidney and liver which are considered to be the principal organs for toxicity assessment. Five fish from each group were analyzed for histological investigations and the obtained results are presented in Figure 10 and Figure 11 (the right side of each panel shows a magnified view).

In the case of kidney morphology, histology of control kidney (Figure 10a) exhibited a normal structure with regular-shaped renal tubules and hematopoietic tissue as depicted with arrows in the magnified view of the sample (Figure 10a, right). On the other hand, kidney sections of NP-exposed fish exhibited various histopathological changes of the normal structure of the tissue. AgNP-treated fish showed epithelial desquamation in renal tubules, edema, and glomerulus shrinkage (Figure 10b). Kidneys of the fish exposed to NiNPs indicated severe vacuolation, necrotic degeneration, and inflammation. The renal tubules structure was completely distorted in the NiNP-treated group showing a complete loss of cellular integrity as illustrated by circle and arrows on the right side of Figure 10c. The epithelial desquamation and vacuolation were observed in kidney samples of Co_3_O_4_NP-treated fish (Figure 8d) while sections of fish exposed to Cr_3_O_4_NPs revealed vacuolation, edema, glomerulus shrinkage, and inflammation in the kidney structure (Figure 10e). The order of renal deformations in kidney samples of NP-treated fish groups was found to be in the following sequence: NiNPs > Cr_3_O_4_NPs > AgNPs > Co_3_O_4_NPs.

Figure 11 shows the histological evaluation of liver sections for control and NP-exposed fish groups. Control liver showed a normal architecture without any pathological abnormalities. A normal morphology of hepatocytes and hepatic parenchyma can be seen in Figure 11a. The liver samples of fish treated with NPs exhibited different alterations in the hepatic tissue. For example, the liver of AgNP-treated fish showed condensed blood vessels (Figure 11b). The liver histology of NiNP-treated fish demonstrated severe blood congestion and vacuolation as shown in Figure 9c while liver sections of Co_3_O_4_NP- and Cr_3_O_4_NP-treated fish groups indicated vacuolation and necrosis Figure 11e,f, respectively. The severity of liver histopathological changes was observed in the following order: NiNPs > Cr_3_O_4_NPs > Co_3_O_4_NPs > AgNPs.

## 4. Discussion

The widespread use of NPs in industries and biomedical applications has raised the need for a comprehensive investigation of their hazardous responses in living organisms.

The toxic effects of different NPs on the organisms depend upon numerous factors including their morphology, size, shape, chemical composition, structural properties, aggregation statue, and surface properties. These factors significantly influence the physiological interactions between NPs and the target tissues [36,37,38]. Different morphologies of NPs such as spherical, triangular, and wire-shaped have different specific lattice structures with high-atom-density facets. These high-atom-density facets provide maximum active surface areas to enhance the reactivity of NPs during the interaction with biological organs. Furthermore, NPs with different morphologies can have different degrees of dispersion, agglomeration, and ion dissolution in the solutions, which result in different levels of induced toxicity [2,39]. Similarly, variation in size of NPs of same material can influence the induced toxic effects significantly. However, the size range for cellular uptake and internalization of NPs into the cells through different pathways including phagocytosis and pinocytosis is up to 100 nm [40].

We performed quantitative and qualitative toxicity assessments on various biological aspects of *L. rohita* using Ag, Ni, Co_3_O_4_, and Cr_3_O_4_ particles of spherical shapes and with sizes in the range of 40 to 60 nm. The degree of toxicity caused by different NPs was found to be varied on different biological parameters. Nanoparticles of different types may exhibit different degrees of toxicity to the same biological system due to various factors such as magnetic or non-magnetic nature, structural composition, and surface chemistry. These factors control physiochemical properties of NPs and strongly affect the interactions between NPs and living tissues to exert their toxic effects [41]. Because of diverse physiochemical properties, different types of NPs express different subcellular localization, cellular uptake, and ability to induce oxidative DNA damage leading to different levels of nanotoxicity [42].

The survival of fish was observed to be unaltered in AgNP-, NiNP-, and Co_3_O_4_NP-treated groups, which suggested their non-acute lethal toxicity while Cr_3_O_4_NPs caused early fish mortalities showing their acute toxic responses (Figure 6a). According to a previous study, AgNPs were proved to be non-lethal to *L. rohita* at concentrations up to 100 mg/kg for a treatment period of 7 days [3], while another study showed their lethal response (LC_50_) to zebrafish even at 3 mg/L concentration for 96 h [43]. In the same study, nickel NPs showed no cumulative mortality to adult zebrafish even at higher concentration > 400 gm/L for 96 h while Boran and Şaffak [44] in their study on larval zebrafish showed acute lethal toxicity (LC_50_) of NiNPs at 122.2 mg/L for 96-h. In the case of cobalt oxide NPs, various studies on zebrafish confirmed the non-lethal response of different types (Co_2_O_3_ and Co_3_O_4_) and shapes (spherical and block) of cobalt oxide NPs even at higher concentrations; 100 mg/L for 24 h, 100 mg/L for 96 h, 200 mg/L for 15 days [45,46,47]. In the case of acute toxicity of Cr_2_O_3_NPs to different aquatic organisms, Tavares et al. [48] observed the mean half-maximal effective concentration (EC50-48h) of 6.74 mg/L for *Daphnia similis* while Puerari et al. [49] obtained the EC_50-48_ h value of 6.79 mg/L for *Daphnia magna* and EC_50-30_ min value of 12.91 mg/L for *Aliivibrio fischeri*. Thus, the results of our and previous studies indicate that lethal concentrations and exposure period may differ for different species. Weight of the fish exposed to NiNPs and Cr_3_O_4_NPs was found to be significantly reduced (Figure 6b). Lower weight gain in fish and mice exposed to metallic (gold) and non-metallic (carbon nanotubes, plastic) NPs has already been reported [50,51,52]. The likely reason for growth reduction could be lack of food digestibility under stressful conditions [52].

Behavioral disturbances were observed in NP-treated fish, which might be due to alterations in fish metabolism and direct transportation of NPs to brain. NPs are reported to damage the texture of fish brain [50]. This damage might be NP type- and concentration-dependent, which leads to different behavioral response in fish treated with different NPs [53,54].

Hemato-immunological responses are regularly employed as markers of physiological stress to toxicants in fish [55,56]. Low TEC and Hb levels can be the indication of anemia and erythropoiesis disorder in NP-treated fish. Zhang et al. [54] found a decreasing trend in TEC and Hct % levels in gold NP-treated murine models at high concentrations. Reduction in TECs, Hb and Hct % were noticed in *Oreochromis niloticus* treated with sub-lethal concentrations of zinc oxide nanoparticle [57]. TLC and Hb concentration was found to be decreased in Fe_2_O_3_NP-treated fish. TEC and Hct % values in silver carp were significantly lowered when exposed to AgNPs for one week [58]. NPs are believed to exert this toxicity by inducing ROS pathway that may destroy membrane integrity [59]. Since TLC and TEC originate from the lymphatic system and hematopoietic tissues, their reduction might indicate the toxic effect of NPs on lymphatic and hematopoietic system, respectively. The suppression in activities of bone marrow stem cell or small life span of TECs could be other possible reasons for low Hb and Hct % [56].

Biomolecules, particularly proteins, are likely to face oxidative damage because of transition metallic ions. Peroxidation or ROS generation may induce oxidative stress leading to variation in these biomolecules. Protein biomarkers are critical indicators of physiological disturbances. Albumin and globulin formulate a major proportion of the body proteins, and alterations occur in their quantities when exposed to xenobiotics. We found a rise in total protein, albumin and globulin in NP-treated fish (Figure 8). The elevated albumin and globulin show a provoked immune response in NP-treated fish. The heightened immune response indicates the weakened and stressful condition of fish, making it vulnerable to diseases. Elevation in total protein and globulin has previously been noted in *Channa punctatus* living in Ni-, Co-, and Cr-polluted water. Although serum albumin decreased significantly in their study, they found a significant increase in its level in liver and muscles [60]. In *Oreochromis niloticus* exposed to Cu and Cd NPs, an elevation in plasma proteins was noted by El-Serafy et al. [61]. Dobsikova et al. [62] noticed an elevation in total protein content in common carp with 7 h transportation stress. An increase in these proteins in NP-treated groups can be assigned to the increased protein synthesis to fulfil the requirements of high energy due to NPs mediated strain and to meet the immunotoxic challenge.

Increase in cholesterol level and higher activities of ALT and AST enzymes were observed in NP-treated groups (Figure 7). ALT and AST are considered to be important parameters for assessing tissue injury or organ dysfunction. Liver is the major organ where these enzymes are generally found, and their increased level shows cellular toxicity [63]. An increase in ALT and AST levels was in accordance with our histopathological results of kidney and liver damage. Monfared et al. [63] also mentioned an increase in cholesterol, ALT, and AST in AgNP-exposed rainbow trout.

The metallic nature of NPs can stimulate an oxidative stress in different organs of animals. The degree of ROS toxicity in various tissues can be function-specific as well as NP composition-specific depending upon the nature of metallic ions release [64,65,66]. Oxidative stress has also been reported by Wise et al. [61] as the causative agent for disintegrating cell membranes and biomolecules. In our work, the treated fish kidney and liver showed remarkable changes in MDA and in antioxidant enzyme (SOD, CAT- and GPx) activities (Figure 9). The reduction in antioxidant enzymes is believed to be caused by NP-induced oxidative stress.

Kidney and liver are the major organs of the body involved in metabolism and clearance of hazardous molecules. When normal architecture of these organs is disturbed by toxicants or other stimulants, all the vital body process may get imbalanced. Their toxicities are basic biosafety evaluation markers for new drugs. Different significant histological alternations in kidney and liver of NP-treated fish revealed the destructive role of NPs, as shown in Figure 10 and Figure 11. These alterations were more severe in NiNP- and Cr_3_O_4_NP-exposed fish as compared to fish samples treated with Co_3_O_4_NPs and AgNPs. The severity of damage caused by different types of NP in kidney and liver tissues can be attributed to the level of their toxicity in exposed fish. Such adverse effects of NPs were also reported by other researchers. Intensified cell apoptosis has been found in head and tail of zebrafish when chronically exposed to high concentration of bulk and TiO_2_NPs [19]. Histopathological alterations were observed in the epidermis, gills, and liver of Siberian sturgeon when exposed to AgNPs and Copper NPs [67]. The incidence of degenerative necrosis in these organs after NPs treatment was found to be associated with oxidative stress, cell membrane and DNA damage, and disorders in the protein, lipid and carbohydrate metabolism [68,69].

## 5. Conclusions

All four types of particles (AgNPs, NiNPs, Co_3_O_4_NPs, and Cr_3_O_4_NPs) with size distribution in the range of 40 to 60 nm caused different degrees of nanotoxicity in *L. rohita* as compared to control (untreated) fish. Variations in different parameters, such as survival rate, growth, behavior, blood parameters, protein, enzyme levels, oxidative stress, and tissue structure deformities, confirmed the toxic effects produced by NPs. Nevertheless, these toxicological impacts were found to be NP type-dependent. AgNPs exhibited least toxicity, while Cr_3_O_4_NPs showed most toxic behavior in our study. The toxicity statistics of this work showed that *L. rohita* can be used as a bioindicator for monitoring the toxic potential of NPs. Furthermore, NP-based elevation or decline in biomolecules can serve as suitable biomarkers for assessing fish health. The outcomes of our work suggest that toxicity of nanoparticles must cautiously be considered before using them in different applications.

## Figures and Tables

**Table 1:** Calculated structural properties of synthesized NPs.

Sample	Peak Position 2*θ* (°)	Diffraction Plane (hkl)	FWHM (Radians)	Crystallite Size, D (nm)	Lattice Constant, a(Ǻ)	Diffraction Plane (hkl)	Crystalline Structure
AgNPs	38.5	111	0.00567	27	4.06	38.5	FCC
NiNPs	44.8	111	0.00521	30	3.168	44.8	FCC
Co_3_O_4_NPs	36.9	311	0.00312	48	8.084	36.9	Cubic Spinal
Cr_3_O_4_NPs	32.7	202	0.00413	37	a = b = 8.72, c = 7.5	32.7	Tetragonal

**Table 2:** Measured magnetic parameters of prepared magnetic nickel and cobalt oxide NPs.

Sample	Saturation Magnetization (Ms, emu/g)	Retentivity (Mr, emu/g)	Coercivity (Hc, Oe)
NiNPs	1.753	0.602	211.96
Co_3_O_4_NPs	0.133	0.0307	157.68

**Table 3:** Behavioral manifestation of *L. rohita* in different experimental groups during the experimental period.

Behavior Categories	Swimming	Interaction	Fin Movement	Feed Intake
Control	Normal swimming	Normal interactions with other fish and environment	Normal fin movements	Normal food intake
AgNPs	Fish movements were quite normal	Normal interactions with other fish and environment	Normal fin movements	Normal food intake
NiNPs	Fish movements were slow, and it also showed jumping	Fish showed avoiding behavior	Slower fin movements	Very low feed intake
Co_3_O_4_NPs	Fast, random movements	Fish showed avoiding behavior	Slower fin movements	Lower feed intake
Cr_3_O_4_NPs	Unrested, random swimming	Fish sometimes showed avoiding behavior and other times more aggressive to other fish	Slower fin movements	Very low feed intake
